# Antifungal activity of *Xenorhabdus* spp. and *Photorhabdus* spp. against the soybean pathogenic *Sclerotinia sclerotiorum*

**DOI:** 10.1038/s41598-020-77472-6

**Published:** 2020-11-26

**Authors:** Julie G. Chacón-Orozco, César Jr. Bueno, David I. Shapiro-Ilan, Selcuk Hazir, Luís G. Leite, Ricardo Harakava

**Affiliations:** 1 Instituto Biológico, APTA, São Paulo, SP 04014-900 Brazil; 2 United States Department of Agriculture, Agricultural Research Service, Southeastern Fruit and 14 Tree Nut Research Laboratory, Byron, GA USA; 3 Department of Biology, Faculty of Arts and Science, Aydin Adnan Menderes University, Aydin, Turkey

**Keywords:** Applied microbiology, Pathogens

## Abstract

The fungus, *Sclerotinia sclerotiorum,* causes white mold disease and infects a broad spectrum of host plants (> 500), including soybean with yield losses of up to 70%. Biological control is a potential alternative for management of this severe plant pathogen, and relative to chemical fungicides, provides broad benefits to the environment, farmers and consumers. The symbiotic bacteria of entomopathogenic nematodes, *Xenorhabdus* spp. and *Photorhabdus* spp., are characterized by the production of antimicrobial compounds, which could serve as potential sources for new bio-fungicides. The objectives of this study were to assess cell-free supernatants (CFS) of 16 strains of these bacteria cultures on *S. sclerotiorum* mycelium growth; assess the volatiles of *X. szentirmaii* cultures on the fungus mycelium and sclerotium inhibition; and evaluate the *X. szentirmaii* cultures as well as their CFS on the protection of soybean seeds against the white mold disease. Among the 16 strains, the CFS of *X. szentirmaii* showed the highest fungicidal effect on growth of *S. sclerotiorum*. The CFS of *X. szentirmaii* inhibited > 98% of fungus growth from mycelium and sclerotia, whereas the volatiles generated by the bacterium culture inhibited to 100% of fungus growth and 100% of sclerotia production. The bacterial culture diluted to 33% in water and coated on soybean seeds inhibited *S. sclerotiorum* and protected soybean plants, allowing 78.3% of seed germination and 56.6% of plant development. Our findings indicate potential for a safe and novel control method for *S. sclerotiorum* in soybean. Moreover, this is the first study to indicate that volatile organic compounds from *Xenorhabdus* spp. can be used in plant disease suppression.

## Introduction

The fungus *Sclerotinia sclerotiorum* that causes white mold disease infects a broad spectrum of host plants (> 500), including soybean with yield losses of up to 70%^1–3^. To control white mold in soybeans, a number of measures are recommended: non-host crops, certified seeds, treatment of seeds with fungicide, uniform coverage of the soil with straw, greater spacing between rows, soil plows, weed control, cleaning of implements and of harvester’s machines, and the use of chemical control by means of foliar spraying, mainly during the period of greatest susceptibility of the soybean plant (beginning of flowering until the beginning of pod formation)^4^. If used in combination, these measures promotes a reduction of inoculum (sclerotia) in the soil and results in increased yields^5^.

Among the control measures listed above, the use of chemical fungicide is the most effective, but it is also the most impactful to the environment and presents hazards to the human health. One alternative to chemical fungicides is the use of biological control, including application of antagonistic fungi and bacteria, as well as their secondary metabolites and volatile organic compounds (VOCs)^6^. Many studies conducted under in vitro conditions have highlighted *Xenorhabdus* spp. and *Photorhabdus* spp. as potential bacteria to control plant diseases^7–10^. The bacteria, *Xenorhabdus* spp. and *Photorhabdus* spp., are symbiotically associated with entomopathogenic nematodes *Steinernema* spp. and *Heterorhabditis* spp., respectively^11^. The bacteria produce several extracellular enzymes^12^, toxins^13^ and antimicrobial substances^14^ that provide protection to the nematodes during their development in the insect host. Several antibiotic and antimycotic compounds produced by *Xenorhabdus* and *Photorhabdus* highlights these bacteria as potential sources for development of new biopesticides^15–24^. *X. bovienii* YL002, *X. nematophila* TB and *X. nematophila* YL001 were the first symbiotic bacteria tested against *S. sclerotiorum* fungus, providing inhibition rates above 91.23 ± 2.67% in vitro conditions^25^. Despite of these results, no study assessed a symbiotic bacteria and their metabolites against *S. sclerotiorum *in vivo conditions. Additionally, and no studies have evaluated VOCs generated by *Xenorhabdus* or *Photorhabdus* cultures against plant pathogenic fungi. The objectives of this study were to assess cell-free supernatants (CFS) of 16 strains of these bacteria cultures on *S. sclerotiorum* mycelium growth; assess the volatiles of *X. szentirmaii* cultures on the fungus mycelium and sclerotium inhibition; and evaluate the *X. szentirmaii* cultures as well as their CFS on the protection of soybean seeds against the white mold disease. The long-term goal is to develop a sustainable alternative for integrated management of white mold disease.

## Results

### Metabolites of *Xenorhabdus* and *Photorhabdus* to inhibit mycelial growth

**Figure 1:**
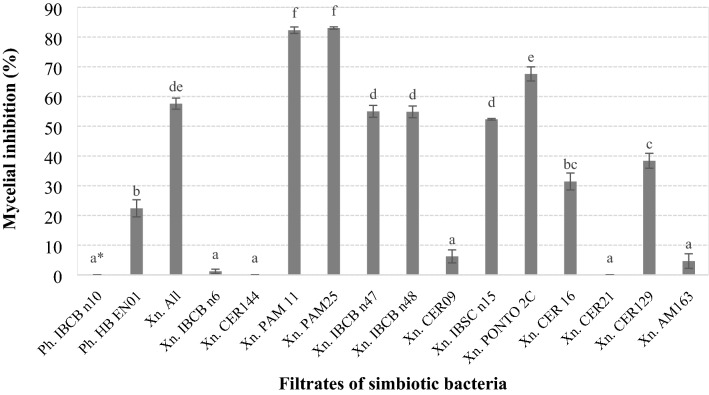
Mycelial growth inhibition (%) of *Sclerotinia sclerotiorum* on solid media containing filtrates of *Photorhabdus* (Ph.) and *Xenorhabdus* (Xn.) strains. Bars with the same letter do not differ statistically by Tukey's test at 5% significance.

Metabolites of the 16 strains of symbiont bacteria varied tremendously in their effect to inhibit mycelial growth of *S. sclerotiorum.* The CFS of the *X. szentirmaii* strains PAM 11 and PAM 25 produced the highest percentages of inhibition on the fungus (82% and 83%, respectively), which differed significantly from the CFS of the other strains (*F* = 304.939; df = 15, 176; *P* < 0.001) (Fig. 1).

The metabolites from *Xenorhabdus* sp., strains IBCB n6, CER144, CER09, CER21 and AM163, and from *P. luminescens* IBCB10, provided the lowest inhibition rates of mycelial growth with variations from 0 to 6% (Fig. 1).

### Metabolites of *Xenorhabdus szentirmaii* to inhibit mycelial growth

**Figure 2:**
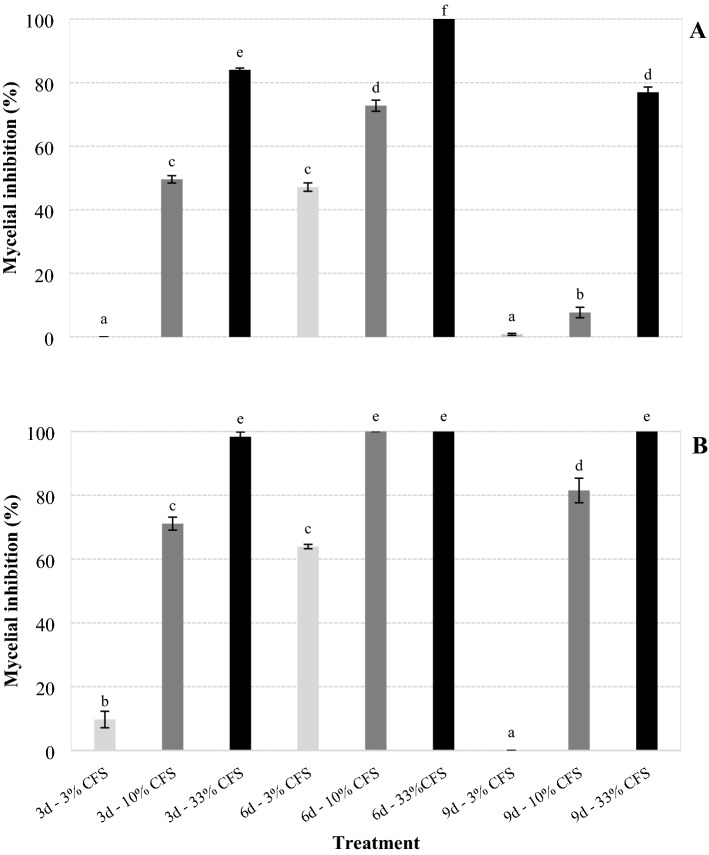
Mycelial growth inhibition (%) of *Sclerotinia sclerotiorum* inoculated as mycelium (**A**) and sclerotium (**B**) on PDA medium containing cell free supernatant (CFS) from cultures of *Xenorhabdus szentirmaii* grown for 3, 6 and 9 days (d) in TSB medium, diluted to 3%, 10% and 33% in water. Bars with the same letter do not differ statistically by Tukey's test at 5% significance.

For the fungus inoculated as mycelium, the CFS of *X. szentirmaii* PAM 25 obtained after 6 days of bacteria growth provided the highest percentages of mycelial inhibition, from 47% (6d—3% CFS) to 100% (6d—33% CFS), which differed significantly in their respective dilutions from CFS obtained after 3 and 9 days of growth (*F* = 899.894; df = 8, 171; *P* < 0.001) (Fig. 2A).

For the fungus inoculated as sclerotium (Fig. 2B), results showed a similar trend to the results obtained when using mycelium as inoculum. However, CFS obtained after 3, 6 and 9 days of bacterial growth, diluted at 33%, and after 6 days diluted at 10%, produced ≥ 98% mycelial inhibition and did not differ from each other (*F* = 522.851; df = 8, 171; *P* = 1.00), but differed in comparison to the other filtrates (*F* = 522.851; df = 8, 171; *P* < 0.001).The filtrate obtained after 6 days of bacterial growth, diluted to 3%, inhibited mycelial growth by 64%, which differed from those obtained after 3 and 9 days of growth in the same dilution (*F* = 522.851; df = 8, 171; *P* < 0.001).

The CFS obtained from cultures grown 3 and 9 days, diluted to 33%, and CFS obtained from cultures grown 6 days, diluted to 10%, did not totally inhibit the mycelial growth of the fungus inoculated as mycelium (Fig. 2A), but totally inhibited the formation of sclerotia that would be produced on the mycelium, even after the mycelium had covered the entire culture medium 20 days post inoculation (Table 1). The other CFS did not totally inhibit the growth of the fungus inoculated as mycelium and sclerotium (Fig. 2a,b), allowing the colonization of the entire culture medium 20 days after inoculation, but caused significant reductions in the number (*F*_mycelium_ = 235.536 and F_sclerotium_ = 379.531; *P* < 0.001; df = 9, 200 for both) and in the diameters (*F*_mycelium_ = 182.312 and F_sclerotium_ = 342, 938; *P* < 0.001; df = 9, 990 for both) of the sclerotia produced, except for the filtrate 3d—3% CFS that did not differ from the control treatment in the number (*F*_mycelium_ = 235.536; *P* = 1.000 and F_sclerotium_ = 379.531; *P* = 0.992; df = 9, 200 for both) and in the diameters (*F*_mycelium_ = 182.312; *P* = 0.329 and F_sclerotium_ = 342, 930; *P* = 1.000; df = 9, 990 for both) of the sclerotia produced (Table 1).

**Table 1:** Number and diameter of sclerotia (± standard error) produced by *Sclerotinia sclerotiorum* inoculated as mycelium and sclerotium on PDA medium containing cell-free supernatant (CFS) from cultures of *Xenorhabdus szentirmaii* grown for 3, 6 and 9 days (d) in TSB medium, diluted to 10 and 33% in water.

Treatment	Number		Diameter (cm)	
	Mycelium	Sclerotium	Mycelium	Sclerotium
3d-3% CFS	24 ± 1.2a	21 ± 0.87a	0.22 ± 0.005ab	0.24 ± 0.008a
3d-10% CFS	13 ± 0.65bc	9 ± 0.6c	0.14 ± 0.015c	0.12 ± 0.005d
3d-33% CFS	0d	0d	0d	0e
6d-3% CFS	17 ± 1.1b	7 ± 0.54c	0.19 ± 0.019b	0.18 ± 0.006b
6d-10% CFS	0d	0d	0d	0e
6d-33% CFS	0d	0d	0d	0e
9d-3% CFS	13 ± 0.75c	12 ± 0.69b	0.13 ± 0.006c	0.20 ± 0.007b
9d-10% CFS	12 ± 1.3c	11 ± 0.37b	0.13 ± 0.008c	0.15 ± 0.011c
9d-33% CFS	0d	0d	0d	0e
Control	24 ± 2.46a	23 ± 1.41a	0.24 ± 0.009a	0.24 ± 0.007a

Different letters in columns indicate differences between treatments (Tukey, *P* < 0.05).

### Antifungal efficacy of VOCs of *Xenorhabdus szentirmaii* against mycelial growth of *S. sclerotiorum*

**Figure 3:**
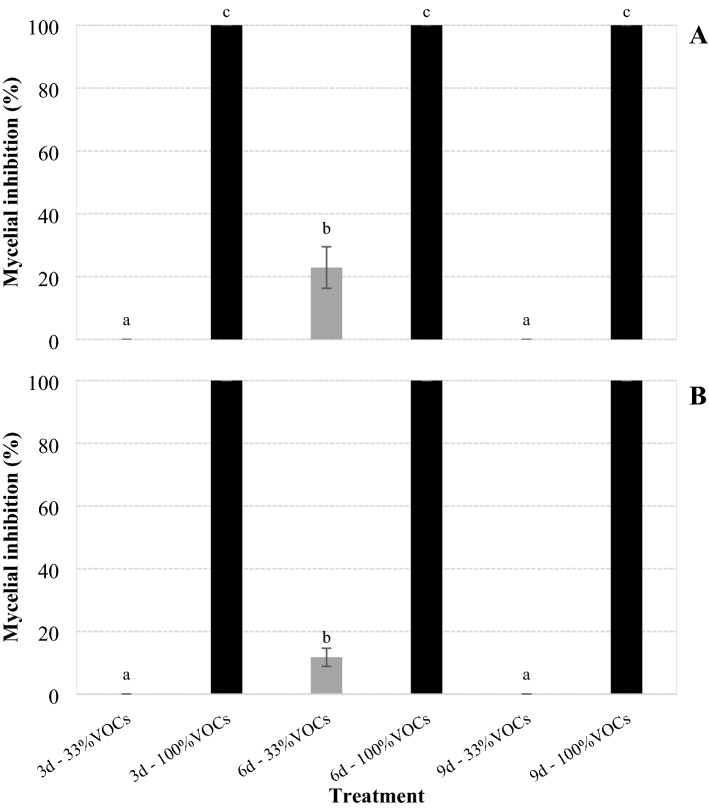
Mycelial growth inhibition (%) of *Sclerotinia sclerotiorum* on solid medium after its exposure as mycelium (**A**) and sclerotium (**B**) to volatiles organic compounds (VOCs) generated by liquid cultures of *Xernorhabdus szentirmaii* PAM 25 grown for 3 and 6 days (d) in TSB, diluted to 33% in water and undiluted (100%). Bars with the same letter do not differ statistically by Tukey's test at 5% significance.

Undiluted VOCs generated by cultures of *X. szentirmaii* PAM 25 grown for 3, 6 and 9 days caused 100% of inhibition of fungus growth after its exposure as mycelium and sclerotium to the VOCs (Fig. 3).

The all 33% diluted VOCs allowed the fungus to grow after its exposition as mycelium and sclerotium (Table 2), with the VOCs generated by cultures with 6 days of bacterial growth providing a slight inhibition (23% from mycelium and 12% from sclerotium), which differs from VOCs generated by cultures with 3 (0%), and 9 (0%) days of bacterial growth (*F*_mycelium_ = 1.180; *F*_sclerotium_ = 1.693; *P* < 0.001; df = 5, 234 for both).

**Table 2:** Number of sclerotia (± standard error) produced by *Sclerotinia sclerotiorum* after its exposure as mycelium and sclerotium to volatiles organic compounds (VOCs) generated by liquid cultures of *Xenorhabdus szentirmaii* PAM 25 grown for 3 and 6 days (d), diluted to 33% in water and undiluted (100%), and inoculated on PDA medium.

Treatment	Mycelium	Sclerotium
3d-33% VOCs	21.3 ± 1.5b	13.75 ± 1.6b
3d-100% VOCs	0e	0d
6d-33% VOCs	5.05 ± 0.8d	0d
6d-100% VOCs	0e	0d
9d-33% VOCs	10.8 ± 0.6c	6.65 ± 1.1c
9d-100% VOCs	0 ± 0 ee	0d
Control H_2_O	31 ± 2a	23 ± 1.5a
Control TSB	31 ± 2a	23 ± 1.5a

Different letters in columns indicate differences between treatments (Tukey, *P* < 0.05).

The VOCs generated by cultures grown 6 days and diluted to 33%, significantly reduced the number of sclerotia produced by the fungus after its exposure as mycelium and inoculation on the medium (Table 2) (*F* = 239.948; df = 7, 152; *P* < 0.001), and totally inhibiting production after its exposition as sclerodium and inoculation on the medium (*F* = 197.825; df = 7, 152; *P* < 0.001). The other VOCs generated by cultures grown 3 and 9 days and diluted to 33% also caused significant reductions in the production of sclerotia (*F*_mycelium_ = 239.948; *F*_sclerotium_ = 197.825; *P* < 0.001; df = 7, 152 for both).

### Metabolites of *Xenorhabdus szentirmaii* to protect soybean seeds

**Figure 4:**
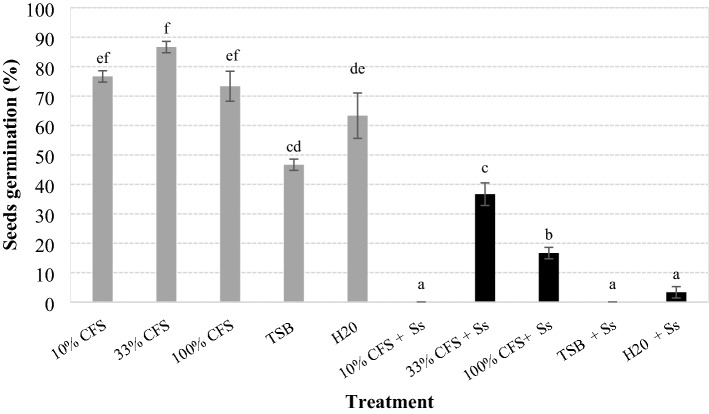
Germination of soybean seeds (%) after being coated with cell-free supernatant (CFS) from cultures of *Xenorhabdus szentirmaii* grown for 6 days in TSB medium, diluted to 10% and 33% in water and undiluted (100%), inoculated or not with *Sclerotinia sclerotiorum* (Ss). Bars with the same letter do not differ statistically by Tukey's test at 5% significance.

The CFS diluted to 33% produced a higher rate of germination (37%) for seeds inoculated with *S. sclerotiorum* (Fig. 4), than the treatments undiluted CFS (17%), CFS diluted to 10% (0%), TSB and H_2_O (5%) (*F* = 90.274; df = 9, 50; *P* < 0.001).

### Cultures of *Xenorhabdus szentirmaii* to protect soybean seeds

**Figure 5:**
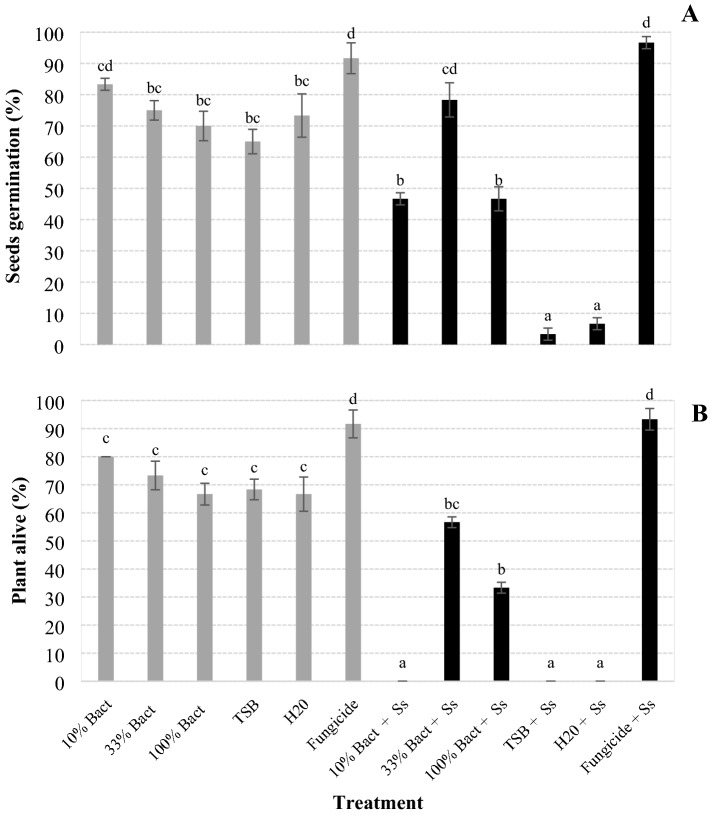
Percentage of germination of soybean seeds (**A**) and of plants alive (**B**) after coating the seeds with the culture of *Xenorhabdus szentirmaii* PAM 25 (Bact) diluted to 10% and 33% in water and undiluted (100%), inoculated or not with *Sclerotinia sclerotiorum* (Ss). Bars with the same letter do not differ statistically by Tukey's test at 5% significance.

The *X. szentirmaii* PAM 25 culture diluted to 33% provided the second highest rate of germination for seeds inoculated with *S. sclerotiorium* (Fig. 5A)*,* and this rate did not differ from the fungicide that provided the highest rate (*F* = 39.712; df = 11, 60; *P* = 0.056), but the treatment did separate from the undiluted filtrate, from 10% dilution, from TSB and H_2_O (*F* = 39.712; df = 11, 60; *P* < 0.001).

The bacterial culture diluted to 33% also provided the second highest rate of survival among plants generated from the seeds inoculated with the fungus (57%) (Fig. 5B), and this rate differed significantly from the fungicide that provided the highest rate (93%), of the bacterial culture diluted to 10%, TSB and from H_2_O, but did not differed from the undiluted culture (*F* = 85.457; df = 11, 60; *P* = 0.130).

**Figure 6:**
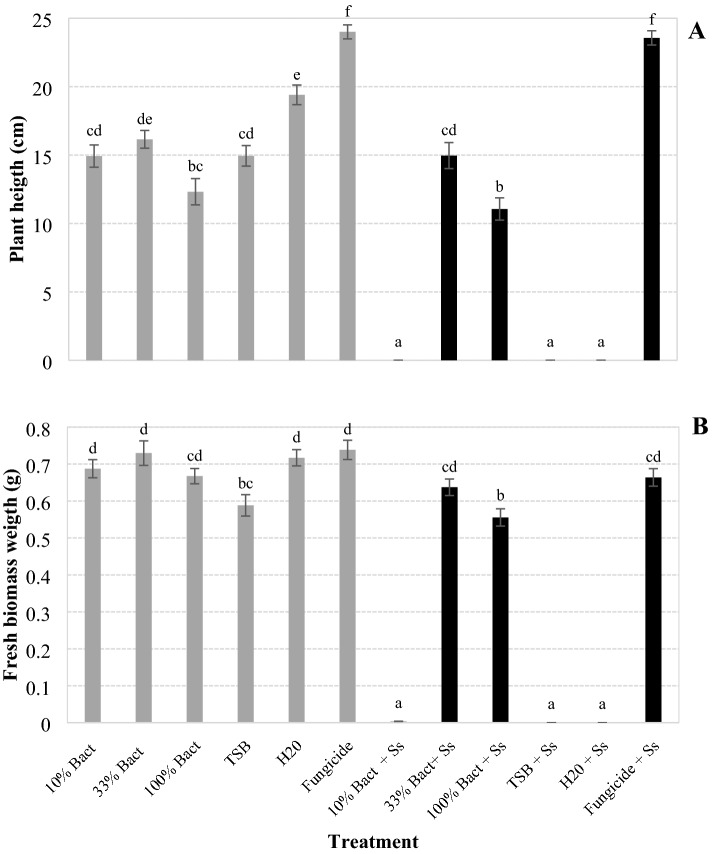
Height (**A**) and weight (**B**) of fresh biomass of soybean plants originated from seeds coated with *Xenorhabdus szentirmaii* PAM 25 cultures, diluted to 10 and 33% in water and undiluted (100%), inoculated or not with *Sclerotinia sclerotiorum* (Ss), 21 days after the treatment. Bars with the same letter do not differ statistically by Tukey's test at 5% significance.

The culture diluted to 33% resulted in the second tallest plants (14.95 cm) among those generated by seeds inoculated with *S. sclerotiorum* (Fig. 6A), and the height differed from the fungicide, which resulted in the tallest plants (23.56 cm), as well as from the undiluted culture (11 cm), the culture diluted to 10% (0 cm), TSB (0 cm), and from H_2_O (0 cm) (F = 164.123; df = 11, 288; *P* < 0.001).

As for biomass (fresh weight of plants), the bacterial culture diluted to 33% resulted in the second highest plant weights (0.63 g) generated by seeds inoculated with *S. sclerotiorum* (Fig. 6B). The plant weight in this treatment (33% with fungus) was not different from the weight in the fungicide treatment (0.66 g) and from all the other treatments (without fungus), except from the four treatments inoculated with the fungus: culture diluted to 10% (0 g), culture undiluted (0.55 g), TSB (0 g) and H_2_O (0 g) (*F* = 189.163; df = 11, 288; *P* > 0.001). Comparing treatments not inoculated with the fungus, TSB medium affect the plant height and weight since their averages were lower compared to those from water.

## Discussion

Our findings on relative potency of the various bacterial metabolites are consistent with previous findings. *X. szentirmaii* stood out as the most promising bacterium to control *S. sclerotiorum*. This species has also stood out in several other studies, with its filtrates inhibiting the growth of several microorganisms, including the bacteria that cause bovine mastitis *Staphylococcus aureus, Escherichia coli and Klebsiella pneumoniae*^7^, the bacteria pathogenic to humans *Escherichia coli, Klebsiella pneumoniae* and *Staphylococcus aureus*, the EPNs symbiont bacteria *Xenorhabdus budapestensis, X. innexi, X. ehlersii, X. nematophila, X. bovienii* and *X. cabanillassii*^9^, the phytopathogenic bacteria *Erwinia amylovora*^8^, and the phytopathogenic fungi *Fusicladium carpophilum, Phytophthora nicotianae, F. effusum, Monilinia fructicola, Glomerella cingulata* and *Armillaria tabescens*^10^. Additionally, in our study, the CFS of the *Photorhabdus* strains provided no more than 22% mycelial inhibition of the fungus, while the CFS of the majority of *Xenorhabdus* isolates provided inhibitions above 30%. Other studies have shown trends of greater antimicrobial bioactivities for *Xenorhabdus* species when compared to *Photorhabdus* species^8,10^. Indeed, these differences in bioactivities among CFS were expected, as the bacteria produce chemical compounds that vary in composition and concentration depending on their species and isolates according to previous studies against other fungi and phytopathogenic bacteria^8,15,27–31^.

We observed higher rates of inhibition for CFS obtained after 6 days of *X. szentirmaii* growth relative to other growth periods tested. This finding may be related to the greater accumulation of secondary metabolites (antiobiotics and exoenzymes). Other studies^12^ showed an increase of exoenzymes (lipases, chitinases, proteases and phospholipases) produced by symbiotic bacteria after the end of the logarithmic phase of growth and lasting until the end of the stationary phase. The lower bioactivities for CFS obtained after 9 days of bacterial growth may be related to the loss of compounds produced by volatilization, as observed in the present study, which may have been accentuated by the constant agitation of the liquid medium in the shaker.

Mycelial growth inhibition increased as the concentration of CFS in the culture medium also increased. This observation held regardless how the fungus was inoculated (mycelium and sclerotium) and the time of bacterial growth. Similar results were observed previously^32^ when assessing the effect of different CFS concentrations (10%, 20%, 40%, 60%, 80% and 100%) obtained from a *X. szentirmaii* (17c + e strain) culture on the growth of the phytopathogenic fungi *Monilinia fructicola* and *Glomerella cingulata*.

Greater mycelial inhibition was caused by the CFS when the fungus was inoculated as sclerotia compared to inoculation as mycelium. Thus, sclerotia were clearly more affected by the CFS than the mycelium discs. This is a surprising result since sclerotia are resistant structures that remain viable for several years in the soil, increasing considerably the difficulties to control the white mold disease^6,33^. Therefore, the inhibition of sclerotia formation or rendering these structures nonviable can interrupt the reproductive cycle of the white mold disease and thereby contribute to controlling the fungus. Some of the bioactive compounds produced by *X. szentirmaii* include xenofuranone A and B^34^, poly-iodinin crystal^35^, szentiamide^36^, xenematide^37^, anthrarufin (anthraquinone)^38^, and fabclavines^39^. All of these compounds have exhibited antimicrobial activity, but their role in suppression of mycelia and sclerotia of *S. sclerotiorum* remains to be studied. Soil microbiota are considered to be the most important factor among those that affect survival of sclerotia in the field, followed by temperature and soil pH^40^.

This study is the first to show the inhibitory effect of VOCs from *X. szentirmaii* cultures on the mycelial growth and sclerotia production of *S. sclerotiorum*. Other studies have assessed the effect of volatiles on the inhibition of *S. sclerotiorum*. However, these prior studies reported on VOCs generated by other bacteria^41–43^ and from fungi^44,45^. Bacteria can interact with fungi by releasing VOCs that can act on a specific fungus or on multiple fungal species in the ecological community^46^. These VOCs cause various levels of impact from almost complete inhibition of mycelial growth, to small growth reductions, and may also cause mycelial and conidial morphological anomalies^47^.

The identification of the volatiles found in the present study may contribute to the development of fungicides with potential as fumigants to treat seeds, soils and substrates contaminated with sclerotia. In another study conducted previously^52^, volatile and non-volatile metabolites of the bacteria *Alcaligenes faecalis* associated symbiotically with nematodes *Oscheius* spp. showed also antifungal activity against the entomopathogenic fungi, *Mucor circinelloides, M. racemosus* and *Rhyzomucor variabilis* (Mucoraceae), as well as against the plant-pathogenic fungus, *Botrytis cinerea* (Helotiales, Sclerotiniaceae). This suggested that *A. faecalis* could benefit *Oscheius* spp. against opportunistic soil-dwelling entomopathogenic fungi, besides protect the plant against phypathogenic fungi. Yet, the study detected a variety of volatile organic compounds produced by *A. faecalis*, including dimethyl disulfide, which were toxic to *R. variabilis* in a dose-dependent manner.

The soybean seeds coated with the *X. szentirmaii* CFS, without the fungus *S. sclerotiorum*, germinated normally, which shows compatibility of the CFS with the plant. Non-toxic effects of the symbiotic bacteria *X. bovienii, X. nematophila, X. cabanillasii, X. szentirmaii, P. temperata, P. luminescens* VS and *P. luminescens* K22 were observed when theirs undiluted filtrates (100%) were sprayed on eggplant (*Solanum melongena* L.), pepper (*Capsicum annuum* L.), tobacco (*Nicotiniana tabacum* L.), tomato (*Solanum lycopersicum* L.), peach (*Prunus persica* L.) and walnuts (*Carya illinoinensis*)^10^. Additional testing may be needed to fully explore the potential phytotoxic effects of *Xenorhabdus* metabolites on various stages of soybeans^10^.

The undiluted CFS (100%) provided less protection to the seeds inoculated with the fungus compared to the CFS diluted to 33% in water, probably due to the higher concentration of TSB medium and not to the higher concentration of secondary metabolites. TSB medium resulted in significantly lower plant height and lower fresh biomass weight compared to H_2_0 when both treatments were tested in the absence of the fungus. Other studies did not show a phytotoxic effect of TSB medium to pecan and peach plants, lettuce seeds as well to seeds and shoots of tomatoes^10,30,31,48,49^. However, these prior studies did not have guargum added to the liquid medium. Thus, the addition of the polysaccharide guargum to the TSB medium in the current study might have affected the plant, probably by maintaining the medium covered on the seed longer time and providing better conditions for the growth of microorganism. In previous study^50^, germination of wheat seeds inoculated with bacteria was either stimulated, or inhibited or remained at control levels depending on the amount of bacteria. The higher the amount of bacteria on plant roots, the smaller was the biomass of plants, but the total photoassimilation was higher. Polysaccharides are usually used for coating seeds with microorganisms in studies of plant protection or plant growth promotion^51^.

Results from the experiments conducted with soybean seeds and plants indicate promise for new fungicidal approaches. The bacterial culture diluted to 33% in water provided protection to seeds inoculated with the fungus similar to that provided by the chemical fungicide in terms of germination and fresh biomass weight of the plants (but not with respect to plant survival and height). The use of bacterial culture could be advantageous relative to the use of its CFS, especially in respect to forgoing the filtration step, a laborious, time consuming and costly operation. New formulations could conceivably improve the efficiency of the culture to make it more potent than the fungicide. Moreover, studies to increase secondary metabolite production and to elucidate active compounds could also lead to new approaches for development of biofungicides. In conclusion, the culture of *X. szentirmaii* diluted to 33% shows potential for the protection of soybean seeds against the fungus *S. sclerotiorum*. Further studies should be conducted to assess the efficiency of the bacterial culture under field conditions.

## Methods

For this study, five experiments were carried out to assess the effects of metabolites from a total of 16 samples of strains of *Xenorhabdus* spp. and *Photorhabdus* spp. on *S. sclerotiorum* mycelium growth; to determine the impact of metabolites and volatiles of *X. szentirmaii* on mycelium and sclerotium; and to assess cultures of *X. szentirmaii* as well as their metabolites on the protection of soybean seeds against the white mold.

### Fungal isolate

The strain of *S. sclerotiorum* used in the tests was isolated from stem of soybean grew in the state of Goiás, Brazil. This isolate belongs to the Phytopathological Collection of the Biological Institute, São Paulo, Brazil, and was supplied by Dr. Silvânia H. Furlan. Before carrying out any test, the pathogenicity of this isolate of *S. sclerotiorum* to soybean plants was confirmed by its inoculation as a mycelium disk on detached leaves of soybean plants (cv. BMX Potência—Embrapa). After its growth on the leaf, the fungus was re-inoculated on potato dextrose agar (PDA) and incubated in a growth chamber for 10 days at 25 °C for mycelial growth, and for an additional 20 days for sclerotia production.

### Bacterial strains and culture conditions

Fourteen strains of *Xenorhabdus* and two of *Photorhabdus* (Table 3) were isolated from entomopathogenic nematodes (EPNs) stored in the collection of the Biological Institute (Campinas-São Paulo-Brazil). For isolation of each bacteria, hemolymph from *Galleria mellonella* (Lepidoptera: Pyralidae) larvae reared on bee wax and previously infected (28–32 h) with the nematode strains was extracted, streaked onto NBTA (nutrient agar 31 g/L, bromothymol blue 25 mg/L, and 2,3,5-triphenyl tetrazolium chloride 40 mg/L), and incubated at 27 °C in the dark according to the methodology described previouly^10,26^. A selected colony from NBTA medium was subcultured to generate a pure *Xenorhabdus* or *Photorhabdus* culture. For long-term storage, the bacteria strains were maintained in Tryptic Soy Broth (TSB) medium supplemented with 20% glycerol, storage at − 80 °C. Before storage, the bacterium culture was checked for its purity based on the morphology and color of its colony on fresh NBTA^10,32^.

**Table 3:** Symbiotic bacteria on the genus *Xenorhabdus* and *Photorhabdus,* and their associated nematodes on the genus *Steinernema* and *Heterorhabditis*, respectively.

Bacteria	Nematode	Strain	Place of the soil source
*X. nematophila*	*S. carpocapsae*	All	USA
*Xenorhabdus* sp.	*S. brazilense*	IBCB n6	Porto Murtinho—MS—Brazil
*Xenorhabdus* sp*.*	*S. brazilense*	PONTO 2C	Porto Murtinho—MS—Brazil
*X. bovienii*	*S. feltiae*	IBCB n47	German
*Xenorhabdus* sp.	*Steinernema* sp.	IBCB n48	Itapetininga—SP- Brazil
*X. doucetiae*	*S. diaprepesi*	IBSC n15	Teodoro Sampaio—SP—Brazil
*X. szentirmaii*	*S. rarum*	PAM 11	Bagé—RS—Brazil
*X. szentirmaii*	*S. rarum*	PAM 25	Bagé—RS—Brazil
*X. romanii*	*S. puertoricense*	CER 09	Rio Verde—GO—Brazil
*X. romanii*	*S. puertoricense*	CER 16	Rio Verde—GO—Brazil
*Xenorhabdus* sp.	*Steinernema sp.*	CER 21	Rio Verde—GO—Brazil
*X. romanii*	*S. puertoricense*	CER 129	Rio Verde—GO—Brazil
*Xenorhabdus* sp.	*Steinernema* sp.	CER144	Rio Verde—GO—Brazil
*X. doucetiae*	*S. diaprepesi*	AM 163	Sinop—MT—Brazil
*P. luminescens*	*H. amazonensis*	IBCB n10	Santa Fé do Sul—SP—Brazil
*P. luminescens*	*H. bacteriophora*	HB EN01	German

### Metabolites of *Xenorhabdus* and *Photorhabdus* to inhibit mycelial growth

This experiment was conducted to assess the effects of metabolites (cell-free supernatants) from 16 strains of *Xenorhabdus* and *Photorhabdus* on the mycelial growth of *S. sclerotiorum*. Methods to assess metabolite activity were based off of those described previously^10,32^. The bacterial strains were confirmed to be in Phase-I by growing them in TSB medium and shaken at 150 rpm and 27 °C for 144 h. The cultures were centrifuged for 60 min (3067.34 RCF, 4 °C) and the supernatants were filter-sterilized (0.22 µm pores)^10,32^. The cell-free supernatants (CFS) were mixed with autoclaved PDA that had been cooled down to about 60 °C, at 10% concentration and, then, poured into Petri-dishes (9 cm)^32^. One plug (0.5 × 0.5 cm) of *S. sclerotiorum* grown for 10 days on PDA was inoculated onto the center of each dish. For each treatment, there were six replicates, with each replication consisting of one dish. For the control, TSB was mixed with PDA at a 10% concentration^32^.

All dishes were incubated at 25 °C in the dark once this fungus is a soilborne phytopathogen and grow in the dark. The colony diameter was recorded daily (cross directions) until the control treatment covered 100% of the medium surface inside the dish. The diameters (cm) of the colonies were transformed to percentages of inhibition growth based on growth in the control treatment [(Mycelial growth in the control − Mycelial growth in the treatment) ÷ Mycelial growth in the control) × 100]. The experiment was conducted twice under the same conditions. The strain that caused the highest mycelial inhibition was selected for the subsequent tests.

### Metabolites of *Xenorhabdus szentirmaii* to inhibit mycelial growth

This experiment was designed to assess the effects of *X. szentirmaii* PAM 25 CFS on *S. sclerotiorum* mycelial growth and sclerotium. The bacteria were grown for 3, 6 and 9 days in TSB, the CFS were mixed with PDA in concentrations of 3, 10 and 30%. Thus, 10 treatments were established, represented by the combinations of the metabolites obtained after three times of bacteria growth and diluted in three concentrations, and by the control (PDA medium**).** The bacteria were grown in three different Erlenmeyer flasks, each containing 300 mL of TSB medium, shaken at 150 rpm and 27 °C, for 3, 6 and 9 days. After each incubation time (3, 6 or 9 days), the bacterial culture was centrifuged and the supernatant was filtered. Then, the CFS obtained was mixed with PDA medium and poured in Petri dishes^10,32^ as mentioned above.

For each treatment, 10 replications were established, each replication consisting of a Petri dish containing PDA medium with or without the CFS. The fungus was inoculated as a mycelium plug according to the methodology described above, and as sclerotium obtained from colonies grown on PDA medium for 20 days in the dark. For both forms of inoculation, the percentage of mycelium inhibition was assessed as previously described, and the number of sclerotia formed per dish was record 20 days after inoculation. The experiment was conducted four times under the same conditions.

### Antifungal efficacy of VOCs of *Xenorhabdus szentirmaii* against mycelial growth of *S. sclerotiorum*

This study evaluated the impact of volatile organic compounds (VOCs) generated by cultures of *X. szentirmaii* PAM 25 on mycelial growth and sclerotia of *S. sclerotiorum*. The bacterium, *X. szentirmaii*, was grown inside Erlenmayer flasks (200 ml) containing 150 ml of TSB medium, shaken at 150 rpm and 27 °C^10,32^. The bacterium was grown for three periods (3, 6 and 9 days). After that the cultures with three growth periods were diluted to 33% in sterile distilled water in a laminar flow chamber or undiluted (100%). Thus, there were eight treatments represented by the combinations of VOCs generated by bacteria cultures grown for three periods, and diluted at 10% or undiluted, and the two control groups water and TSB.

**Figure 7:**
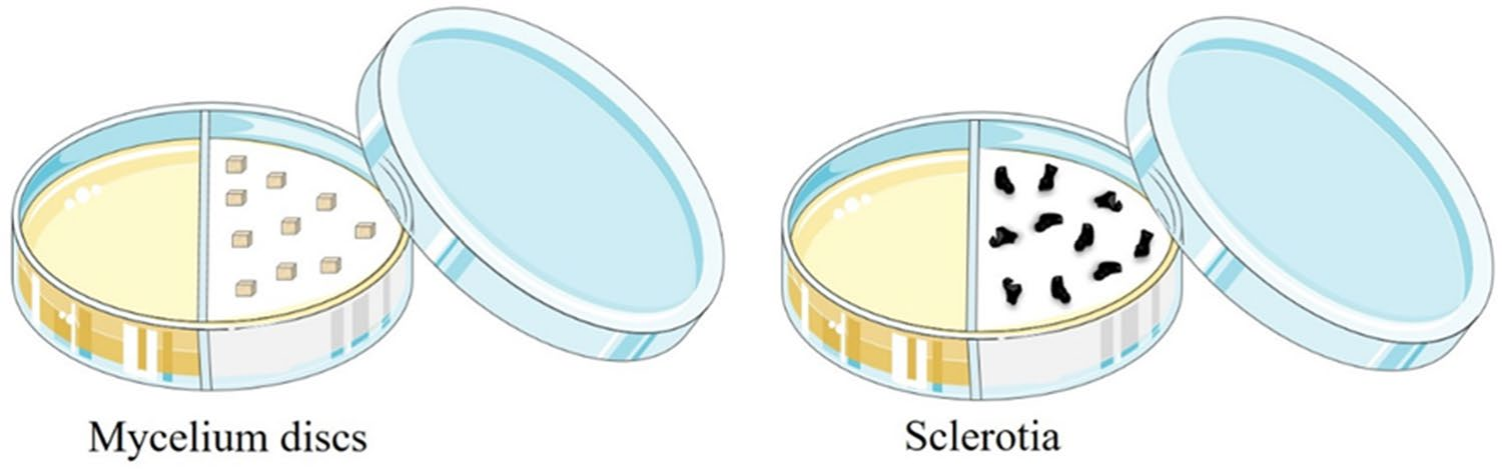
Two partitioned Petri dish containing the bacteria culture on one side, and on the other side 10 discs of mycelium or 10 sclerotia of *S. sclerotiorum* for their exposition to the volatile organic compounds.

For each treatment, 10 replications were established. Each replication consisted of a sterile two partitioned Petri dish: one compartment containing the bacteria culture, and the other containing 10 discs of mycelium (obtained after 10 days of growth in PDA, at 25 °C, in the dark), or 10 sclerotia of *S. sclerotiorum* (obtained after 20 days of growth in PDA, at 25 °C, in the dark) (Fig. 7). To avoid the splash of culture to the opposite side of the dish during transport or handling, the TSB liquid medium with the bacterium as well as the controls TSB and water were thickened by the addition of 0.6% agar before their autoclavation and their use for the experiment. The dishes were capped and sealed with parafilm to prevent loss of the volatiles produced by the bacteria, and then incubated for 14 days at 25 °C in the dark. Subsequently, the mycelium discs and sclerotia were removed and inoculated individually in the center of Petri dishes (9 cm) containing PDA medium. Fungal growth was assessed as previously described. For the plates inoculated with *S. sclerotiorum* mycelium discs, evaluation was carried out 4 days after inoculation. For plates inoculated with sclerotia, evaluations were made after 20 days. The experiment was conducted four times under the same conditions.

### Metabolites of *Xenorhabdus szentirmaii* to protect soybean seeds

This study assessed the effects of *X. szentirmaii* PAM 25 metabolites on the protection of soybean seeds from *S. sclerotiorum.* The bacterium was grown in Erlenmeyer flasks containing 300 mL of TSB medium and shaken at 150 rpm and 27 °C for 6 days. The bacterial culture was centrifuged at 3067.34 RCF (3800 rpm) for 2 h and then filtered as previously described. The obtained CFS was diluted in sterile distilled water and tested in 3 different concentrations: 10%, 33% and 100% (undiluted). The metabolites were thickened with 1% guar gum and then fixed and coated on soybean seeds (variety BMX Potência—Embrapa). Distilled water and TSB medium thickened with guargum (1%) were used as control. Thus, there were 10 treatments represented by CFS diluted to 10% and 33% in water, and undiluted (100%), coated on soybean seeds and, then, inoculated or not with *S. sclerotiorum*, and by two control treatments with water and TSB medium on the seeds inoculated or not with the fungus. The coated seeds were distributed individually over filter papers moistened with sterile distilled water (2 mL/plate) in Petri dishes. For the seeds with *S. sclerotiorum*, each seed was inoculated with a fungus mycelium disk obtained after 10 days of growth in PDA at 25 °C in the dark. The dishes were sealed with PVC film and incubated at 25 °C for 7 days in the dark.

There were three replications per treatment with each replication consisting of a Petri dish containing 10 seeds. Evaluation was carried out 7 days after the experiment was initiated by counting the number of germinated and non-germinated seeds. The test was conducted twice under the same conditions.

### Cultures of *Xenorhabdus szentirmaii* to protect soybean seeds

This experiment was carried out to determine the effects of *X. szentirmaii* PAM25 cultures on the protection of soybean seeds against *S. sclerotiorum*. Treatments were established at the same manner as described in the previous study but for this time the chemical fungicide Certeza (Methyl Thiophanate + Fluazinam) was included. The methodology used was the same as described in the previous study, except that the coated seeds were distributed individually in plastic cups (1 L), over cotton soaked with sterile distilled water (200 mL). The pots were sealed with PVC film (with three small openings) and incubated at 25 °C and 12 h of photoperiod for 21 days. For each treatment, three replications were established with each replication consisting of a plastic pot containing ten seeds. Evaluations were carried out after 7 days by counting the number of germinated seeds, after 14 days by counting the number of live plants, and after 21 days, by measuring fresh biomass and plant length (root + shoot). The test was conducted twice under the same conditions.

### Statistical analyzes

Data were analyzed using the General Linear Model (GLM). Means were compared at the *P* = 0.05 level, and Tukey’s Honestly Significant Difference (Tukey HSD) test was used to separate means (SPSS,2011). Percentage of growth inhibition, seeds germination and live plants were arcsine transformed (√x/100) before analysis^10,53^. Data on the number of sclerotia, diameter of sclerotium, height and fresh weight of soybean plants were square-root transformed (√X + 0.5). Non-transformed means are presented in the results.

## References

[1] Purdy LH (1979). *Sclerotinia sclerotiorum*: history, diseases and symptomatology, host range, geographic distribution, and impact. Phytopathology.

[2] Bolton ML, Thomma BPHJ, Nelson BD (2006). *Sclerotinia sclerotiorum* (Lib.) de Bary: biology and molecular traits of a cosmopolitan pathogen. Mol. Plant Pathol..

[3] Saharan GS, Mehta N (2008). Sclerotinia Diseases of Crop Plants: Biology, Ecology and Disease Management.

[4] Peltier AJ (2012). Yield loss and control of Sclerotinia stem rot of soybean. J. Integr. Pest Manag..

[5] Görgen CA, Silveira Neto AN, Carneiro LC, Ragagnin V, Lobo Júnior M (2009). Controle do mofo branco com palhada e *Trichoderma harzianum* 1306 em soja. Pesq. Agrop. Bras..

[6] Fernando WGD, Ramarathnam R, Krishnamoorthy AS, Savchuk SC (2005). Identification and use of potential bacterial organic antifungal volatiles in biocontrol. Soil Biol. Biochem..

[7] Furgani G (2008). *Xenorhabdus* antibiotics: a comparative analysis and potential utility for controlling mastitis caused by bacteria. J. Appl. Microb..

[8] Böszörménvi E (2009). Isolation and activity of *Xenorhabdus* antimicrobial compounds against the plant pathogens *Erwinia amylovora* and *Phytophthora nicotianae*. J. Appl. Microbiol..

[9] Fodor A (2010). Comparative analysis of antibacterial activities of *Xenorhabdus* species on related and nonrelated bacteria *in vivo*. J. Microb. Antimicrob..

[10] Hazir S (2016). Relative potency of culture supernatants of *Xenorhabdus* and *Photorhabdus* spp. on growth of some fungal phytopathogens. Eur. J. Plant Pathol..

[11] Shapiro-Ilan DI, Hazir S, Glazer I, Lacey LA (2017). Basic and applied research: entomopathogenic nematodes. Microbial Control of Insect and Mite Pests, From Theory to Practice.

[12] Forst S, Nealson K (1996). Molecular biology of the symbiotic-pathogenic bacteria *Xenorhabdus* spp. and *Photorhabdus* spp. Microbiol. Rev..

[13] Ensign, J. C. et al. Proteins from the genus *Xenorhabdus* are toxic to insects on oral exposure. US patent. No. 0147148 A1. (2002).

[14] Akhurst RJ (1982). Antibiotic activity of *Xenorhabdus* spp. bacteria symbiotically associated with insect pathogenic nematodes of the families Heterorhabditidae and Steinernematidae. J. Gen. Microbiol..

[15] Paul VJ, Frautschy S, Fenical W, Nealson KH (1981). Antibiotics in microbial ecology, isolation and structure assignment of several new antibacterial compounds from the insect symbiotic bacteria *Xenorhabdus* spp. J. Chem. Ecol..

[16] Richardson WH, Schmidt TM, Nealson KH (1988). Identification of an anthrquinona pigmet and a hydroxystilbene antibiotic from *Xenorhabdus luminescens*. Appl. Environ. Microbiol..

[17] McInerney B (1991). Biologically active metabolites from *Xenorhabdus* spp., Part 1. Dithiolopyrrolone derivatives with antibiotic activity. J. Nat. Prod..

[18] McInerney BV, Taylor WC, Lacey MJ, Akhurst RJ, Gregson RP (1991). Biologically active metabolites from *Xenorhabdus* spp., part 2. Benzopyran-1-one derivatives with gastroprotective activity. J. Nat. Prod..

[19] Akhurst RJ, Dunphy GB, Beckage N, Thompson S, Federici B (1993). Tripartite interactions between symbiotically associated entomopathogenic bacteria, nematodes and their insect hosts. Parasites and Pathogens of Insects.

[20] Chen G (1996). Chitinase activity of *Xenorhabdus* and *Photorhabdus* species, bacterial associates of entomopathogenic nematodes. J. invertebr. Pathol..

[21] Li JJ, Chen G, Webster JM (1997). Nematophin, a novel antimicrobial substance produced by *Xenorhabdus nematophilus* (Enterobactereaceae). Can. J. Microbiol..

[22] Ji D, Yi Y, Kim Y (2004). 16S rDNA sequence and biochemical characters of a Korean isolate of *Xenorhabdus nematophila*. J. Asia Pac. Entomol..

[23] Lang G, Kalvelage T, Peters A, Wiese J, Imhoff JF (2008). Linear and cyclic peptides from the entomopathogenic bacterium *Xenorhabdus nematophilus*. J. Nat. Prod..

[24] Gualtieri M, Aumelas A, Thaler J-O (2009). Identification of a new antimicrobial lysine-rich cyclolipopeptide family from *Xenorhabdus nematophila*. J. Antibiot..

[25] Fang XL, Li ZZ, Wang YH, Zhang X (2011). *In vitro* and *in vivo* antimicrobial activity of *Xenorhabdus bovienii* YL002 against *Phytophthora capsici* and *Botrytis cinerea*. J. Appl. Microb..

[26] Akhurst RJ (1980). Morphological and functional dimorphism in *Xenorhabditis* spp., bacteria symbiotically associated with the insect pathogenic nematodes Neoaplectana and Heterorhabditis. J. Gen. Microbiol..

[27] Chen G, Dunphy GB, Webster JM (1994). Antifungal activity of two *Xenorhabdus* species and *Photorhabdus luminescens*, bacteria associated with the nematodes *Steinernema* species and *Heterorhabditis megidis*. Biol. Control..

[28] Isaacson PJ, Webster JM (2002). Antimicrobial activity of *Xenorhabdus* sp. RIO (Enterobacteriaceae), symbiont of the entomopathogenic nematode, *Steinernema riobrave* (Rhabditida: Steinernematidae). J. invertebr. Pathol..

[29] Webster JM, Gaugler R (2002). Bacterial metabolites. Entomopathogenic Nematology.

[30] Shapiro-Ilan DI, Reilly CC, Hotchkiss MW (2009). Suppressive effects of metabolites from *Photorhabdus* and *Xenorhabdus* spp. on phytopathogens of peach and pecan. Arch. Phytopathol. Plant Prot..

[31] Shapiro-Ilan DI, Bock CH, Hotchkiss MW (2014). Suppression of pecan and peach pathogens on different substrates using *Xenorhabdus bovienii* and *Photorhabdus luminescens*. Biol. Control.

[32] Hazir S, Shapiro-Ilan D, Bock CH, Leite LG (2018). Thermo-stability, dose effects and shelf-life of antifungal metabolite-containing supernatants produced by *Xenorhabdus szentirmaii*. Eur. J. Plant Pathol..

[33] Ferraz LCL, Bergamin Filho A, Amorim L, Nasser LCB (2003). Viabilidade de *Sclerotinia sclerotiorum* após a solarização do solo na presença de cobertura morta. Fitopatol. Bras..

[34] Brachmann AO (2006). Xenofuranones A and B: phenylpyruvate dimers from *Xenorhabdus szentirmaii*. J. Nat. Prod..

[35] Fodor A, Ehlers RU, Enkerli J, Glazer I, Ferber ML, Tkaczuk C (2008). New aspects on *Xenorhabdus* antibiotics research. Insect Pathogens and Insect Parasitic Nematodes.

[36] Nollmann FI (2012). Synthesis of szentiamide, a depsipeptide from entomopathogenic *Xenorhabdus szentirmaii* with activity against *Plasmodium falciparum*. Beilstein J. Org. Chem..

[37] Ohlendorf B, Simon S, Wiese J, Imhoff JF (2011). Szentiamide, an *N*-formylated cyclic depsipeptide from *Xenorhabdus szentirmaii* DSM 16338T. Nat. Prod. Commun..

[38] Ladell, P. *Isolation and Characterization of Antibiotics Produced by the Nematode Symbiont**Xenorhabdus szentirmaii.* 70 p. Thesis MSc. in Biology. (University of Wisconsin, USA, 2011).

[39] Fuchs SW, Grundmann F, Kurz M, Kaiser M, Bode HB (2014). Fabclavines: bioactive peptide–polyketide–polyamino hybrids from *Xenorhabdus*. ChemBioChem.

[40] Adams PB, Ayers WA (1979). Ecology of *Sclerotinia* species. Phytopathology.

[41] Liu W-W, Mu W, Zhu B-Y, Du Y-C, Liu F (2008). Antagonistic activities of volatiles from four strains of *Bacillus* spp. and *Paenibacillus* spp. against soilborne plant pathogens. Agric. Sci. China.

[42] Wu Y (2015). Effects of volatile organic compounds from *Streptomyces albulus* NJZJSA2 on growth of two fungal pathogens. J. Basic Microb..

[43] Giorgio A, De Stradis A, Lo Cantore P, Iacobellis NS (2015). Biocide effects of volatile organic compounds produced by potential biocontrol rhizobacteria on *Sclerotinia sclerotiorum*. Front. Microb..

[44] Lobo M, Abreu MS (2000). Inibição do crescimento micelial de *Sclerotinia sclerotiorum* por metabólitos voláteis produzidos por alguns antagonistas em diferentes temperaturas e pH's. Ciênc. Agrot..

[45] Fialho MB, Moraes MHD, Tremocoldi AR, Pascholati SF (2011). Potential of antimicrobial volatile organic compounds to control *Sclerotinia sclerotiorum* in bean seeds. Pesq. Agropec. Bras..

[46] Kai M (2009). Bacterial volatiles and their action potential. Appl. Microb. Biotechnol..

[47] Chaurasia B (2005). Diffusible and volatile compounds produced by an antagonistic *Bacillus subtilis* strain cause structural deformations in pathogenic fungi *in vitro*. Microbiol. Res..

[48] Carvalho DDC (2007). Rhizobacteria able to produce phytotoxic metabolites. Braz. J. Microbiol..

[49] Shurigin V, Davranov K, Wirth S, Egamberdieva D, Bellingrath-Kimura SD (2018). Medicinal plants with phytotoxic activity har-bour endophytic bacteria with plant growth inhibitory properties. Environ. Sustain..

[50] Somova LA, Pechurkin NS, Sarangova AB, Pisman TI (2001). Effect of bacterial population density on germination wheat seeds and dynamics of simple artificial ecosystems. Adv. Space Res..

[51] O’Callaguan M (2016). Microbial inoculation of seed for improved crop performance: issues and opportunities. Appl. Microbiol. Biotechnol..

[52] Shan S (2019). The symbiotic bacteria *Alcaligenes faecalis* of the entomopathogenic nematodes *Oscheius* spp. exhibit potential biocontrol of plant and—entomopathogenic fungi. Microb. Biotechnol..

[53] Cevizci D (2020). Mode of entry of secondary metabolites of the bacteria *Xenorhabdus szentirmaii* and *X. nematophila* into *Tetranychus urticae*, and their toxicity to the predatory mites *Phytoseiulus persimilis* and *Neoseiulus californicus*. J. Invertebr. Pathol..

